# Gender differences among patients with drug resistant tuberculosis and HIV co-infection in Uganda: a countrywide retrospective cohort study

**DOI:** 10.1186/s12879-021-06801-5

**Published:** 2021-10-24

**Authors:** Joseph Baruch Baluku, David Mukasa, Felix Bongomin, Anna Stadelmann, Edwin Nuwagira, Sabine Haller, Kauthrah Ntabadde, Stavia Turyahabwe

**Affiliations:** 1 Division of Pulmonology, Kiruddu National Referral Hospital, Kampala, Uganda; 2 Makerere University Lung Institute, PO Box 26343, Kampala, Uganda; 3 Complex Diseases and Genome Epidemiology, Graduate School of Public Health, Seoul National University, Seoul, South Korea; 4 Department of Medical Microbiology & Immunology, Faculty of Medicine, Gulu University, Gulu, Uganda; 5 Division of Epidemiology and Community Health, School of Public Health, University of Minnesota, Minneapolis, MN USA; 6 Infectious Diseases Unit, Department of Medicine, Mbarara University of Science and Technology, Mbarara, Uganda; 7 Department of Public and Global Health, Epidemiology, Biostatistics, & Prevention Institute, University of Zurich, Zurich, Switzerland; 8 MRC/UVRI & LSHTM Uganda Research Unit, Entebbe, Uganda; 9 National Tuberculosis and Leprosy Control Program, Ministry of Health, Kampala, Uganda

**Keywords:** Gender differences, TB/HIV, MDR, Mortality, Men, Women, Drug resistance, Tuberculosis, Sex

## Abstract

**Background:**

Gender differences among patients with drug resistant tuberculosis (DRTB) and HIV co-infection could affect treatment outcomes. We compared characteristics and treatment outcomes of DRTB/HIV co-infected men and women in Uganda.

**Methods:**

We conducted a retrospective chart review of patients with DRTB from 16 treatment sites in Uganda. Eligible patients were aged ≥ 18 years, had confirmed DRTB, HIV co-infection and a treatment outcome registered between 2013 and 2019. We compared socio-demographic and clinical characteristics and tuberculosis treatment outcomes between men and women. Potential predictors of mortality were determined by cox proportional hazard regression analysis that controlled for gender. Statistical significance was set at p < 0.05.

**Results:**

Of 666 DRTB/HIV co-infected patients, 401 (60.2%) were men. The median (IQR) age of men and women was 37.0 (13.0) and 34.0 (13.0) years respectively (p < 0.001). Men were significantly more likely to be on tenofovir-based antiretroviral therapy (ART), high-dose isoniazid-containing DRTB regimen and to have history of cigarette or alcohol use. They were also more likely to have multi-drug resistant TB, isoniazid and streptomycin resistance and had higher creatinine, aspartate and gamma-glutamyl aminotransferase and total bilirubin levels. Conversely, women were more likely to be unemployed, unmarried, receive treatment from the national referral hospital and to have anemia, a capreomycin-containing DRTB regimen and zidovudine-based ART. Treatment success was observed among 437 (65.6%) and did not differ between the genders. However, mortality was higher among men than women (25.7% vs. 18.5%, p = 0.030) and men had a shorter mean (standard error) survival time (16.8 (0.42) vs. 19.0 (0.46) months), Log Rank test (p = 0.046). Predictors of mortality, after adjusting for gender, were cigarette smoking (aHR = 4.87, 95% CI 1.28–18.58, p = 0.020), an increase in alanine aminotransferase levels (aHR = 1.05, 95% CI 1.02–1.07, p < 0.001), and history of ART default (aHR = 3.86, 95% CI 1.31–11.37, p = 0.014) while a higher baseline CD4 count was associated with lower mortality (aHR = 0.94, 95% CI 0.89–0.99, p = 0.013 for every 10 cells/mm^3^ increment).

**Conclusion:**

Mortality was higher among men than women with DRTB/HIV co-infection which could be explained by several sociodemographic and clinical differences.

## Background

Tuberculosis (TB) is the leading cause of death among people living with HIV (PLHIV) accounting for 208,000 HIV-related deaths globally in 2019 [1]**.** The incidence of TB among PLHIV has steadily declined both globally and in sub-Saharan Africa (SSA) due to an increase in antiretroviral therapy (ART) coverage [2, 3]. However, the emergence of drug resistant tuberculosis (DRTB) is a threat to TB control. Globally, there were over 465,000 incident cases of rifampicin resistant TB in 2019 and treatment success was reported among 57% [1]. A meta-analysis reported treatment success of multi-drug resistant TB (MDRTB)—resistance of *Mycobacterium tuberculosis* (*Mtb*) to rifampicin and isoniazid—among adult PLHIV to be only 50% [4]. In SSA, where more than 70% of PLHIV are found [5], the treatment success in DRTB/HIV co-infection is 45% despite a high uptake of ART [6]. This is significantly lower in PLHIV than HIV uninfected individuals with MDRTB (risk ratio = 0.87) [6].

Gender differences in TB socio-demographic and biological risk factors, case notification rates and treatment outcomes are well recognised in literature [7, 8]. In 2019, 56% of global TB cases were among men [1]. Among people with DRTB (regardless of HIV status), being a man has been reported to be a predictor of poor treatment outcomes in Korea [9], South Africa [10] and Georgia [11], while being a woman predicted treatment success in Russia [12]. However, being a woman was reported to be a predictor of extensively drug resistant tuberculosis (MDRTB with additional resistance to fluroquinolones and injectable second line aminoglycoside) independent of HIV infection in South Africa [13]. Socio-demographic and clinical gender differences are not well documented among DRTB/HIV co-infected patients.

Characterising gender differences in DRTB/HIV co-infection is important in SSA where women have a disproportionally higher prevalence of HIV and a higher risk of TB/HIV-related mortality than men [14–17]. Identifying gender differences can guide implementation of gender-specific interventions to improve DRTB treatment outcomes. Uganda is a TB/HIV high burdened country, and 52–62% of patients with DRTB are co-infected with HIV [18, 19]. In this study, we compared socio-demographic and clinical characteristics and treatment outcomes of DRTB/HIV co-infected men and women in Uganda, drawn from a large countrywide retrospective cohort [20]. We further determined predictors of mortality in this population.

## Methods

All methods used in the study were carried out in accordance with relevant guidelines and regulations.

### Study setting and population

This was a countrywide retrospective cohort of patients with DRTB/HIV co-infection in Uganda. We reviewed patients’ charts and treatment registers from 16 DRTB treatment sites in Uganda. Eligible patients had laboratory confirmed DRTB and HIV co-infection and a documented treatment outcome between 2013 and 2019. We excluded all DRTB patients with missing treatment charts, those who were not initiated on DRTB therapy, patients with DRTB whose therapy was later changed to first line agents and those for whom a treatment outcome was reported as “not evaluated”. There are 17 DRTB treatment sites in Uganda, comprising of 1 urban national referral hospital in the capital city (Kampala), 13 rural regional referral hospitals and 3 rural district hospitals. We were unable to collect data from one regional referral hospital (Gulu Regional Referral Hospital with 160 potential participants) due to COVID-19 travel restrictions at the time of data accrual. A full description of DRTB management in Uganda are described elsewhere [20, 21]. Briefly, there were three changes to the treatment guidelines of DRTB during the period under study. In 2012, the guidelines recommended a standardised treatment regimen comprised of 6 months of kanamycin (Km) (or capreomycin), levofloxacin (Lfx), ethionamide (Eto), cycloserine (Cs), and pyrazinamide (Z) followed by 18 months without the aminoglycoside [22]. Ethambutol (E), amikacin and p-amino salicylic acid were recommended as alternative agents. In 2016, an intensive phase of Km + Lfx + Eto + Cs + Z for 6 months or 4 months after culture conversion (whichever was longer) was recommended [23]. Thereafter, a 14-months’ (or at least 20 months after culture conversion) continuation phase without the aminoglycoside was recommended. In 2017, an annex to the guidelines introduced a short-term regimen for patients whose DRTB was sensitive to an injectable aminoglycoside and fluroquinolones. This comprised of 4–6 months of Km + moxifloxacin (Mfx) + clofazimine (Cfz) + Z + E + Isoniazid (H)^high dose^ + Eto and 5 months of Mfx + Cfz + Z + E [24]. Bedaquiline became progressively available by 2018 but was recommended by the national DRTB consilium on a case-by-case basis [25].

### Study measurements

Socio-demographic characteristics were extracted from the patients’ charts using a data abstraction form. Specifically, age, gender, residence (rural or urban), employment and marital status, any history of cigarette or alcohol use, level of DRTB treatment initiation health facility (district hospital, regional referral hospital and national referral hospital), year of DRTB treatment initiation and history of TB treatment were extracted.

HIV-related clinical variables extracted were the baseline CD4 T-cell count at the time of HIV diagnosis, status of HIV viral load suppression (suppression defined as < 1000 copies/ml) at the time of DRTB treatment initiation, ART status at DRTB treatment initiation, duration of ART use prior to DRTB therapy, drugs in the ART regimen, documented history of ART-default and use of cotrimoxazole prophylaxis. ART default was defined as a patient who had not visited the health facility HIV clinic in 3 or more consecutive months at any point in their care since they initiated ART [26]. Other clinical variables were TB drug resistance profiles at baseline, other comorbid conditions, number and type of drugs in the DRTB treatment regimen, duration from DRTB confirmation to DRTB treatment initiation, month of culture conversion, and duration of DRTB treatment. The average haemoglobin, liver aminotransferase and creatinine levels in the first 6 months of therapy was also extracted for each patient. Treatment outcomes (cure, treatment completion, death and loss-to-follow-up) were defined according to the World Health Organisation’s definitions [27]. Specifically, treatment success was a sum of cure and treatment completion. Mortality was defined as death from any cause during DRTB treatment. Other study procedures are described elsewhere [20].

### Statistical analysis

Data were analysed using SAS version 9.4 (SAS Inc., Cary, NC, USA). Categorical socio-demographic and clinical variables were summarised as proportions and compared between men and women using Pearson’s Chi-square test and Fisher’s exact test. Continuous variables were summarised as medians with the corresponding interquartile range, and were compared between men and women using the Two Sample Kolmogorov–Smirnov Test. We used Kaplan Meier curves to assess the survival differences between men and women. Further, we used Cox proportional-hazard regression models to assess predictors of mortality. We tested the assumptions underlying Cox proportional hazards model and assessed the functional form of covariates. To develop a parsimonious model, we used hierarchical cluster analysis and assessed estimated coefficients for predictors in the univariate analysis to select representative predictors for each cluster of correlated variables. The predictors were retained in the model if they were significant at α = 0.1 using a backward selection procedure. While gender predicted mortality at bivariable analysis, it was eliminated during backward selection of variables for the multivariable model. We, therefore, intentionally kept in the model to control for its possible influence despite having α > 0.1. For all analyses we performed complete case analysis and statistical significance was set at p < 0.05.

## Results

### Participant enrolment

**Fig. 1:**
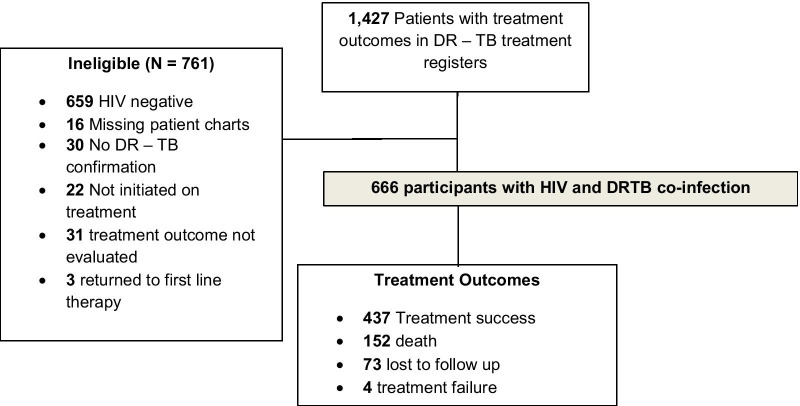
Study flow diagram

Participants’ charts were reviewed between January and March 2020. A total of 1427 charts were reviewed and 666 patients were eligible for the study. The study flow diagram is shown in Fig. 1.

### Baseline characteristics of the study population

Of 666 patients, 401 (60.2%) were men. The median (IQR) age of the entire population was 36.0 (13) years. Among the patients, 622 (95.0%) were on ART, 587 (97.4%) were on cotrimoxazole prophylaxis and the baseline median (IQR) CD4 count (n = 190) was 179.5 (311.0) cells per mm^3^. Viral load suppression (n = 68) was reported among 47 (69.1%). Eight (3.0%) women were pregnant at the start or during DRTB treatment. Other characteristics of patients with DRTB/HIV co-infection are shown in Tables 1 and 2.

**Table 1:** Gender differences in socio-demographic of patients with DRTB/HIV co-infection

Characteristic	Total population	Men n = 401	Women n = 265	p-value
DRTB treatment site, n = 666				**0.006**
National referral hospital	210 (31.5)	108 (26.9)	102 (38.5)	
Regional referral hospital	404 (60.7)	258 (64.3)	146 (55.1)	
District hospital	52 (7.8)	35 (8.7)	17 (6.4)	
Age, median (IQR), years	36.0 (13.0)	37.0 (13.0)	34.0 (13.0)	**< 0.001**
Age (years), n = 643				**0.007**
< 15	11 (1.71)	6 (1.53)	5 (1.98)	
15–34	264 (41.06)	140 (35.81)	124 (49.21)	
35–60	355 (55.21)	236 (60.36)	119 (47.22)	
> 60	13 (2.02)	9 (2.30)	4 (1.59)	
Residence, n = 640				0.059
Rural	392 (61.25)	246 (64.23)	146 (56.81)	
Urban	248 (38.75)	137 (35.77)	111 (43.19)	
Employment status, n = 637				**< 0.001**
Unemployed	170 (26.69)	72 (18.85)	98 (38.43)	
Self employed	305 (47.88)	185 (48.43)	120 (47.06)	
Formal employment	162 (25.43)	125 (32.72)	37 (14.51)	
Marital status, n = 632				**< 0.001**
Married	303 (47.94)	208 (55.17)	95 (37.25)	
Not married	329 (52.06)	169 (44.83)	160 (62.75)	
History of TB treatment, n = 666				0.060
Yes	359 (53.90)	228 (56.86)	131 (49.43)	
No	307 (46.10)	173 (43.14)	134 (50.57)	
Year of treatment initiation, n = 660				0.591
≤ 2013	54 (8.18)	32 (8.08)	22 (8.33)	
2014	71 (10.76)	38 (9.60)	33 (12.50)	
2015	103 (15.61)	68 (17.17)	35 (13.26)	
2016	143 (21.67)	83 (20.96)	60 (22.73)	
2017	136 (20.61)	80 (20.20)	56 (21.21)	
2018	109 (16.52)	71 (17.93)	38 (14.39)	
2019	44 (6.67)	24 (6.06)	20 (7.58)	
Alcohol use, n = 464				**< 0.001**
Yes	183 (39.44)	143 (51.62)	40 (21.39)	
No	281 (60.56)	134 (48.38)	147 (78.61)	
Cigarette smoking, n = 464				**< 0.001**
Yes	90 (19.40)	82 (29.60)	8 (4.28)	
No	374 (80.60)	195 (70.40)	179 (95.72)	

Bolded p-values indicate a statistically significant result

*DRTB* drug resistant tuberculosis, *TB* tuberculosis, *IQR* interquartile range

**Table 2:** Gender differences in clinical characteristics of patients with DRTB/HIV co-infection

Characteristic	Total population	Men, n = 401	Women, n = 265	p-value
Type of DRTB at baseline, n = 666				**< 0.001**
Rifampicin resistance	441 (66.2)	253 (63.1)	188 (70.9)	
MDRTB	203 (30.5)	134 (33.4)	69 (26.0)	
Poly resistant tuberculosis (TB)	13 (2.0)	9 (2.2)	4 (1.5)	
Pre-XDRTB	7 (1.1)	5 (1.3)	2 (0.8)	
XDR-TB	2 (0.3)	0 (0.0)	2 (0.8)	
Resistance at baseline (n = 612)				
Isoniazid	208 (34.0)	138 (37.6)	70 (28.6)	**0.021**
Streptomycin	119 (19.4)	81 (22.1)	38 (15.5)	**0.045**
Ethambutol	103 (16.8)	68 (18.5)	35 (14.3)	0.169
Pyrazinamide	8 (1.3)	3 (0.9)	5 (2.0)	0.124
Aminoglycoside	6 (1.0)	4 (1.1)	2 (0.8)	0.289
Fluroquinolone	3 (0.5)	1 (0.3)	2 (0.8)	0.345
No. of drugs patients was resistant to median (IQR)	1.0 (1.0)	1.0 (1.0)	1.0 (1.0)	0.189
Drugs in treatment regimen				
Kanamycin, n = 665	594 (89.32)	358 (89.50)	236 (89.06)	0.856
Levofloxacin, n = 665	556 (83.6)	337 (84.3)	219 (82.6)	0.583
Ethambutol, n = 612	103 (16.83)	68 (18.53)	35 (14.3)	0.169
Clofazimine, n = 664	113 (17.0)	69 (17.3)	44 (16.6)	0.817
High dose Isoniazid, n = 664	109 (16.4)	65 (16.3)	44 (16.6)	0.915
Capreomycin, n = 664	77 (11.6)	37 (9.3)	40 (15.1)	**0.022**
Ethionamide/Prothionamide, n = 664	659 (99.3)	396 (99.3)	263 (99.3)	0.347
Cycloserine, n = 664	544 (81.9)	327 (82.0)	217 (81.9)	0.982
Bedaquilline	18 (2.71)	10 (2.50)	8 (3.02)	0.687
Linezolid	9 (1.36)	6 (1.50)	3 (1.13)	1.000
Amikacin	5 (0.75)	3 (0.75)	2 (0.75)	1.000
Pyrazinamid	8 (1.31)	3 (0.82)	5 (2.04)	0.124
High-dose isoniazid	208 (33.99)	138 (37.60)	70 (28.57)	**0.021**
Moxifloxacin	107 (16.09)	62 (15.50)	45 (16.98)	0.611
p-Amino salicylic acid	8 (1.20)	4 (1.00)	4 (1.51)	0.233
Total drugs in regimen, n = 666				0.211
≤ 5	394 (59.2)	245 (61.1)	149 (56.2)	
> 5	272 (40.8)	156 (38.9)	116 (43.8)	
Time to treatment initiation, median (IQR), days, n = 649	9.0 (19.0)	9.0 (18.0)	8.0 (19.0)	0.894
Time to culture conversion (months), median (IQR)	3.0 (4.0)	2.0 (4.5)	4.0 (4.0)	0.475
Total treatment duration (months), median (IQR)	20.0 (13.4)	20.0 (14.3)	20.0 (12.0)	0.995
Baseline body mass index, median (IQR), kg/m^2^	18.1 (4.5)	18.0 (4.1)	18.4 (5.3)	0.999
Hearing loss, n = 459	196 (42.7)	112 (41.3)	84 (44.7)	0.475
Cancer, n = 666	11 (1.7)	8 (2.0)	3 (1.1)	0.540
Diabetes, n = 69	20 (29.0)	12 (30.0)	8(27.4)	0.827
Psychiatric symptoms or mental illness n = 666	34 (5.1)	16 (4.0)	18 (6.8)	0.108
Previous exposure to second line drugs n = 666	18 (2.7)	12 (3.0)	6 (2.3)	0.571
Number of poor prognostic indicators, median (IQR)	3.0 (2.0)	3.0 (2.0)	3.0 (2.0)	0.124
ART use, n = 655	622 (95.0)	374 (95.2)	248 (94.7)	0.771
History of ART default, n = 491	86 (17.5)	53 (17.7)	33 (17.2)	0.878
Viral load, n = 68				0.822
Suppressed	47 (69.1)	26 (70.3)	21 (67.7)	
Non-suppressed	21 (30.9)	11 (29.7)	10 (32.3)	
Baseline CD4 Counts, median (IQR), n = 190	179.5 (311.0)	197.0 (317.0)	155.0 (286.0)	0.773
Cotrimoxazole prophylaxis, n = 603	587 (97.4)	351 (97.2)	236 (97.5)	0.828
Drug in the ART regimen				
Emitricitabine, n = 595	11 (1.9)	6 (1.7)	5 (2.1)	0.760
Dolutegravir, n = 595	14 (2.4)	10 (2.8)	4 (1.7)	0.398
Lopinavir, n = 595	9 (1.5)	5 (1.4)	4 (1.7)	0.760
Nevirapine, n = 595	73 (12.3)	37 (10.3)	36 (15.3)	0.067
Tenofovir, n = 595	460 (77.3)	290 (80.6)	170 (72.3)	**0.019**
Zidovudine, n = 594	121 (20.4)	61 (16.9)	60 (25.6)	**0.010**
Years on ART, median (IQR)	3.0 (4.5)	3.0 (4.0)	3.0 (5.0)	0.314
AST (U/l), median (IQR), n = 541	39.3 (30.0)	41.3 (29.0)	36.3 (26.4)	**0.046**
ALT (U/l), median (IQR), n = 541	16.0 (20.3)	16.8 (21.1)	15.8 (19.2)	0.356
GGT (U/l), median (IQR), n = 229	63.0 (87.8)	81.5 (89.0)	55.5 (66.6)	**0.016**
ALP (U/l), median (IQR), n = 308	132.8 (109.2)	136.0 (122.6)	127.0 (97.5)	0.088
Total bilirubin (mg/dl), median (IQR), n = 463	0.318 (0.339)	0.35 (0.37)	0.28 (0.31)	**0.004**
Creatinine (μmol/l), median (IQR), n = 543	70.2 (32.4)	75.9 (31.9)	64.3 (25.7)	**< 0.001**
Haemoglobin (g/dl), median (IQR), n = 580	12.1 (3.3)	12.6 (3.2)	11.7 (3.5)	**< 0.001**
Anemia				**< 0.001**
Normal haemoglobin level	197 (33.97)	144 (41.62)	53 (22.65)	
Mild	186 (32.07)	99 (28.61)	87 (37.18)	
Moderate	149 (25.69)	82 (23.70)	67 (28.63)	
Severe	48 (8.28)	21 (6.07)	27 (11.54)	

Bolded p-values indicate a statistically significant result

*AST* aspartate aminotransferase, *GGT* gamma-glutamyl aminotransferase, *ALT* alanine aminotransferase, *ALP* alkaline aminotransferase, *ART* antiretroviral therapy, *MDRTB* Multi-drug resistant tuberculosis, *XDRTB* extensively drug resistant tuberculosis

### Socio-demographic gender differences among patients with DRTB/HIV co-infection

The median (IQR) age of men and women was 37.0 (13) and 34.0 (13) years respectively (p < 0.001). Men were more likely to have history of cigarette (p < 0.001) and alcohol use (p < 0.001) than women. Women were more likely to be unemployed (p < 0.001), unmarried (p < 0.001), and to receive DRTB treatment from the national referral hospital (p = 0.006). Other socio-demographic differences are shown in Table 1.

### Gender differences in clinical characteristics of patients with DRTB/HIV co-infection

Men were more likely to have MDRTB (p < 0.001), Isoniazid (p = 0.021) and Streptomycin resistance (p = 0.045) and had higher median (IQR) creatinine (75.9 (31.9) vs. 64.3 (25.7) μmol/l**,** p < 0.001), serum aspartate aminotransferase (41.3 (29.0) vs. 36.3 (26.4) U/l, p = 0.046), gamma-glutamyl aminotransferase (81.5 (89.0) vs. 55.5 (66.6) U/l, p = 0.016) and total bilirubin levels (0.35 (0.37) vs. 0.28 (0.31) mg/dl p = 0.004) than women. Additionally, men were more likely to be on Tenofovir-based ART (p = 0.019) and high-dose Isoniazid-containing DRTB regimen (p = 0.021). Women had a lower median (IQR) haemoglobin level (11.7 (3.5) vs. 12.6 (3.2) mg/dl, p < 0.001) and were more likely to have anaemia (p < 0.001), Zidovudine-based ART (p = 0.010) and Capreomycin-containing DRTB regimen (p = 0.022) than men. Other clinical differences are shown in Table 2. There was no difference in the time from diagnosis to treatment initiation between the genders.

### Gender differences in the treatment outcomes of DRTB/HIV co-infection

**Fig. 2:**
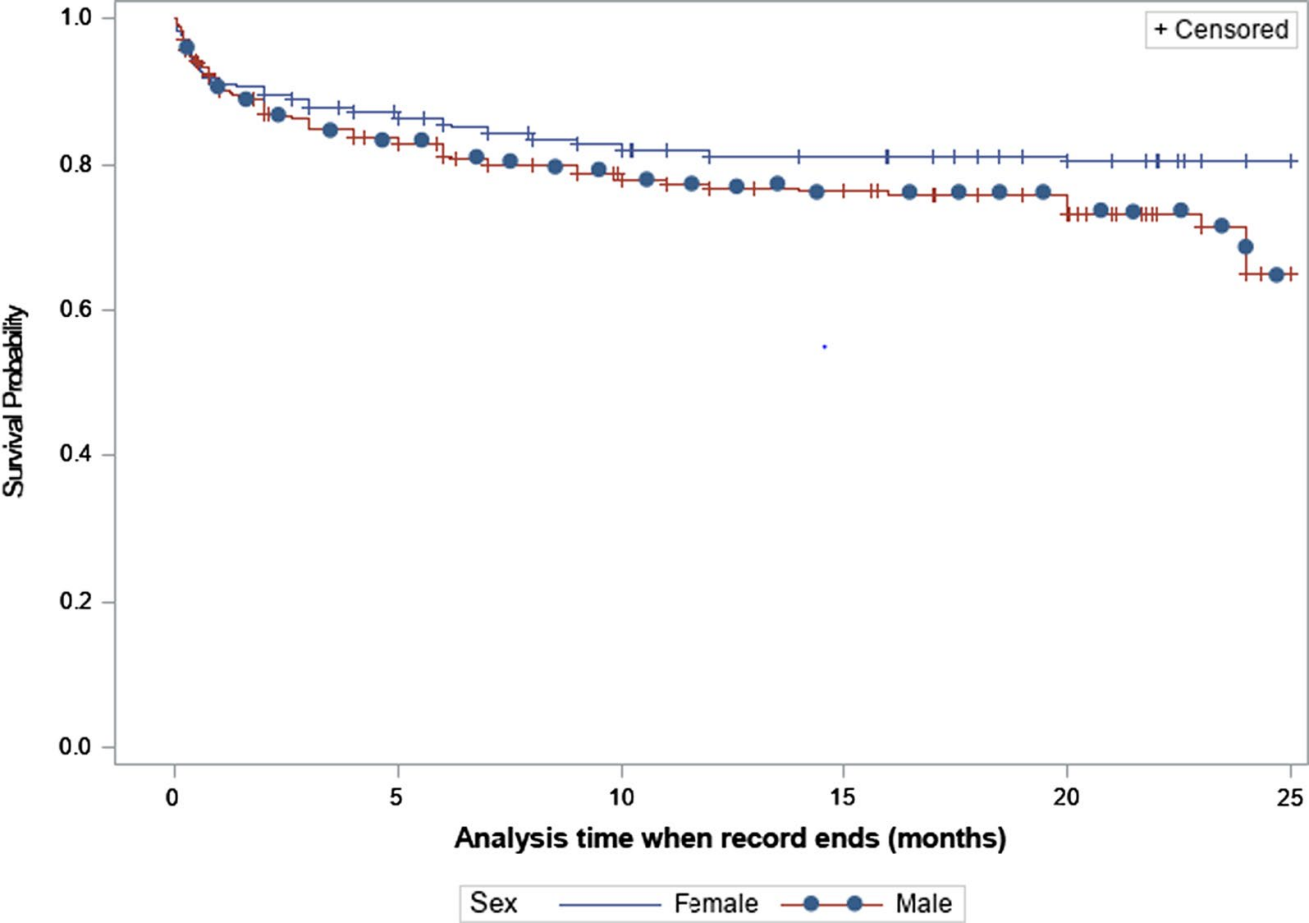
Kaplan Meier curves showing survival differences in men and women with DRTB/HIV co-infection

Men had a higher mortality than women (25.7% vs. 18.5%, p = 0.030) and shorter mean (standard error) survival time (16.8 (0.42) vs. 19.0 (0.46) months), Log Rank test (p = 0.046) (Fig. 2). There was no difference in treatment success, loss to follow up and treatment failure between men and women respectively. Table 3 shows the comparison in treatment outcomes between men and women.

**Table 3:** Gender differences in the treatment outcomes among patients with DRTB/HIV co-infection

Treatment outcome	Total population N = 666	Men n = 401	Women n = 265	p-value
Mortality, n = 666				**0.030**
Yes	152 (22.82)	103 (25.69)	49 (18.49)	
No	514 (77.18)	298 (74.31)	216 (81.51)	
Treatment completion and cure, n = 666				0.064
Success	437 (65.62)	252 (62.84)	185 (69.81)	
No success	229 (34.38)	149 (37.16)	80 (30.19)	
Treatment loss to follow up, n = 666				0.809
Yes	73 (10.96)	43 (10.72)	30 (11.32)	
No	593 (89.04)	358 (89.28)	235 (88.68)	
Treatment failure				1.000
Yes	4 (0.60)	3 (0.75)	1 (0.38)	
No	662 (99.40)	398 (99.25)	264 (99.62)	

Bolded p-values indicate a statistically significant result

### Predictors of mortality among patients with DRTB/HIV co-infection

As shown in Table 4, after adjusting for gender, predictors of mortality were cigarette smoking (aHR = 4.87, 95% CI 1.28–18.58, p = 0.020), an increase in ALT levels (aHR = 1.05, 95% CI 1.02–1.07, p < 0.001), and history of ART default (aHR = 3.86, 95% CI 1.31–11.37, p = 0.014) while higher baseline CD4 count was associated with lower risk of mortality (aHR = 0.94, 95% CI 0.89–0.99, p = 0.013 for every 10 cells/mm^3^ increment). Compared to the year 2012, participants enrolled in 2018 had a higher risk for mortality (aHR: 2.33, 95% CI 1.10–4.93, p = 0.027), adjusted for age and gender. Enrolment in any other year did not predict mortality.

**Table 4:** Parsimonious model for predictors of mortality among DRTB patients with HIV co-infection

Characteristic	aHR	95% CI		p-value
Gender				
Women	1			
Men	1.365	0.422	4.421	0.6036
Cigarette smoking				
None	1			
Yes	4.873	1.278	18.58	**0.0204**
Alanine aminotransferase	1.047	1.023	1.072	**0.0001**
Number of drugs in DRTB regimen				
< 5 drugs	1			
≥ 5 drugs	0.272	0.071	1.038	0.0568
History of ART default				
No	1			
Yes	3.863	1.313	11.365	**0.0141**
Baseline CD4 count (per 10 cells/mm^3^)	0.935	0.887	0.986	**0.0132**

Bolded p-values indicate a statistically significant result

## Discussion

In this study we compared the socio-demographic and clinical characteristics and treatment outcomes among men and women with DRTB/HIV co-infection in Uganda. We found that men were significantly more likely to be on Tenofovir-based ART and to have history of cigarette or alcohol use. They were also more likely to have MDRTB, Isoniazid and Streptomycin resistance, and had higher creatinine, ALT, GGT and total bilirubin levels. Conversely, women were more likely to be unemployed, unmarried, receive treatment from the national referral hospital and to have anemia, capreomycin-containing DRTB regimen and zidovudine-based ART. Mortality was higher among men, although gender did not predict mortality at multivariable analysis. Overall, cigarette smoking, history of ART default and higher ALT levels predicted a higher risk of mortality while higher baseline CD4 counts were associated with lower risk, independent of the gender.

The ratio of women to men with DRTB/HIV co-infection was 1:1.5. This is consistent with the burden of TB by gender (women to men ratio of 1.1.5) in low and middle income countries [28]. To explain this bias, our study found that cigarette smoking and alcohol use, which are known risk factors for DRTB (and drug sensitive TB as well) [29, 30], were mostly prevalent among men. Moreover, history of TB treatment, another risk factor for DRTB was observed mostly among men although the difference was marginally statistically significant (p = 0.060). Smoking cessation and reducing alcohol use may promote adherence to first-line TB therapy and reduce the incidence of DRTB among men.

Similar to our findings, men with TB were more likely to be older than women in a study from Portugal [31]. It is likely that lifestyle exposures (smoking and alcohol use)—exposures whose risk is cumulative with age—are the key drivers of TB risk among men, while HIV infection is the main risk factor for women [32]. In Uganda, the prevalence of HIV among women in reproductive age is almost twice that of men in the same age group [33]. Our study identified unemployment and being unmarried as social inequalities faced by women which have been reported to drive the HIV epidemic among women in Uganda [14]. Interestingly, being unmarried and having a low income have also been identified as risk factors for primary DRTB in China [34]. In our study, women tended (p = 0.060) to have primary DRTB, although this was not statistically significant. It is plausible that unemployment and being unmarried can result in poor health seeking behaviour which can promote community transmission of primary DRTB. However, our results did not suggest differences in delay from diagnosis to treatment initiation. An evaluation of differences in income, social support and health seeking behaviour among women and men with DRTB/HIV co-infection would have complimented the characterisation of the social inequalities. More women than men were urban residents, and this could explain why they mostly received treatment from the national referral hospital, an urban facility, compared to other facilities that are located in rural districts of Uganda.

Our findings show a disproportionately high frequency of risk factors for poor DRTB outcomes among men with HIV/DRTB. Studies have reported cigarette smoking [35], alcohol use [36], MDRTB [37], and elevated creatinine [38], liver transaminases and bilirubin [39] to predict worse outcome among DRTB patients. Moreover, even other risk factors for poor outcomes such as rural residence [40] and history of TB treatment [41] were observed mostly among men, although the difference had marginal statistical significance. Among these, cigarette smoking, alcohol use, liver injury (elevated liver enzymes and bilirubin) and elevated creatinine levels are modifiable factors that could improve treatment outcomes if intervened upon. Smoking cessation [42] and reducing alcohol consumption [43] are interventions that improve TB outcomes. Higher levels of creatinine and liver injury observed among men in our study can be explained by several factors. First, men were more likely to be on a TDF-containing ART regimen. TDF has been associated with elevated creatinine due to renal tubular dysfunction and elevated ALP because of its osteoblastic activity [44]. It is therefore desirable that patients with DRTB/HIV co-infection on TDF-containing ART should not be prescribed second-line injectable agents due to synergistic renal toxicity [45]. The change to all oral DRTB regimens will hopefully reduce such ART and DRTB therapy drug-drug interactions. Secondly, as discussed above, more men had a history of alcohol consumption which causes liver injury that manifests as elevated liver enzymes and bilirubin [46]. Lastly, more men were prescribed high-dose isoniazid than women, which increases liver enzymes by a direct effect of activated isoniazid and its intermediate drug metabolites (hydrazine and acetylhydrazine) [47]. Keshavjeel et al. [39] reported elevated creatinine, liver enzymes and bilirubin to be associated with hepatotoxicity among patients with MDRTB in Russia. Considering that an elevation in ALT was associated with mortality in our study, the need for monitoring for hepatic injury cannot be over emphasised especially in patients with other risk factors for liver injury (such as alcohol use and renal insufficiency). The lower haemoglobin level and anaemia among women in our study can be attributed to AZT-based ART which was mostly prescribed among women. AZT inhibits proliferation of red blood-cell progenitor cells and this risk has been reported to be higher among women [48]. Additionally, the majority of the women were in reproductive age in which menstrual blood loss is associated with negative iron balance [49]. Taken together, our findings re-emphasize the need for designing and adapting ART and DRTB regimens that are customised to each patient’s risk factors for drug adverse events. The evolving landscape of ART and DRTB regimens will need continuous surveillance and documentation of drug-drug interactions to guide future recommendations for optimising both therapies. It is unclear why more men were prescribed TDF-based ART while more women received AZT-based ART. This warranties further investigation. However, this distribution of ART regimens has been previously reported by Castelnuovo and colleagues in Uganda [50].

Considering the high frequency of risk factors for poor DRTB treatment outcomes among men, it is clear why men had higher mortality in our study. Men with HIV and DRTB co-infection have been reported to have higher mortality than women in South Africa as well [51]. The ART and cotrimoxazole coverage was uniform between the genders in our study. Additionally, the ART adherence, baseline CD4 and viral load suppression were comparable although the absolute numbers were small. Therefore, it is likely that the difference in mortality between the genders is due to the poor prognostic indicators as discussed above. This is supported by our multivariate model which demonstrated that gender was not significantly associated with higher risk of mortality in the presence of other risk factors. Only cigarette smoking, ART default and baseline CD4 predicted mortality. Low baseline CD4 counts have been reported to be associated with higher odds of mortality among DRTB/HIV co-infected patients in South Africa [52]. CD4 T-lymphocytes produce interferon-gamma which is important in immune responses against TB [53]. Low CD4 counts are therefore associated with globally impaired TB immune responses and severe TB disease [54]. It should be expected that ART default will be associated with a failure to reconstitute the immune system. Strategies to improve ART adherence such as integrated ART and DRTB care would facilitate ART adherence and improve DRTB outcomes.

Our study has some limitations. Firstly, the retrospective nature of the study did not allow a detailed collection of the required data to achieve a complete characterisation of the patients. Specifically, the counts for viral load and CD4 were too few and this may have affected our ability to detect differences in these variables. During the period under study, HIV and DRTB care was not integrated, and participants received care for either disease at separate health facilities. It is therefore not surprising that these data were not consistently documented at the DRTB treatment site. Secondly, HIV treatment has significantly improved over the study period from the period when the CD4 count was used to determine eligibility for ART initiation to the “test and treat” era. Additionally, there have been several changes in DRTB treatment recommendations in the last 7 years. However, the year of enrolment into care was not associated with higher risk of mortality when adjusted for gender. While patients enrolled in 2018 had higher risk of mortality, this is likely due to documentation bias of deaths since majority of patients would not be expected to have completed treatment by 2019. However, the strength of our study is in its inclusiveness of patients from multiple sites across the country and thus reflective of treatment outcomes in the study population nationwide. Our study provides baseline data to inform policy and prospective studies exploring gender-targeted therapies in this population.

## Conclusion

Men had more risk factors of mortality than women. Cigarette smoking cessation and improving ART adherence could potentially reduce the risk of mortality among DRTB/HIV co-infected individuals. ART and DRTB regimens need to be adapted to patients’ risk for adverse drug events for which monitoring should be performed with fidelity.

## Data Availability

Datasets used in this analysis are available from the corresponding author on reasonable request.

## Abbreviations

TB: Tuberculosis
DRTB: Drug resistant tuberculosis
MDRTB: Multi-drug resistant tuberculosis
XDRTB: Extensively drug resistant tuberculosis
SLDs: Second-line drugs
SSA: Sub-Saharan Africa
RR: Rifampicin resistance
DST: Drug susceptibility test
WHO: World Health Organisation
IQR: Interquartile range
SD: Standard deviation
CI: Confidence interval
AST: Aspartate aminotransferase
ALT: Alanine aminotransferase
ART: Antiretroviral therapy
GGT: Gamma-glutamyl transferase
PLHIV: People living with HIV

## References

[1] World Health Organisation (2020). Global tuberculosis report.

[2] Kyu HH, Maddison ER, Henry NJ, Ledesma JR, Wiens KE, Reiner R (2018). Global, regional, and national burden of tuberculosis, 1990–2016: results from the Global Burden of Diseases, Injuries, and Risk Factors 2016 Study. Lancet Infect Dis.

[3] Dye C, Williams BG (2019). Tuberculosis decline in populations affected by HIV: a retrospective study of 12 countries in the WHO African Region. Bull World Health Organ.

[4] Isaakidis P, Casas EC, Das M, Tseretopoulou X, Ntzani EE, Ford N (2015). Treatment outcomes for HIV and MDR-TB co-infected adults and children: systematic review and meta-analysis. Int J Tuberc Lung Dis.

[5] Kharsany ABM, Karim QA (2016). HIV infection and AIDS in Sub-Saharan Africa: current status. Chall Oppor Open AIDS J.

[6] Chem ED, Van Hout MC, Hope V (2019). Treatment outcomes and antiretroviral uptake in multidrug-resistant tuberculosis and HIV co-infected patients in Sub Saharan Africa: a systematic review and meta-analysis. BMC Infect Dis.

[7] Nhamoyebonde S, Leslie A (2014). Biological differences between the sexes and susceptibility to tuberculosis. J Infect Dis.

[8] Karim F, Islam MA, Chowdhury AMR, Johansson E, Diwan VK (2007). Gender differences in delays in diagnosis and treatment of tuberculosis. Health Policy Plan.

[9] Jeon DS, Shin DO, Park SK, Seo JE, Seo HS, Cho YS (2011). Treatment outcome and mortality among patients with multidrug-resistant tuberculosis in tuberculosis hospitals of the public sector. J Korean Med Sci.

[10] Elliott E, Draper HR, Baitsiwe P, Claassens MM (2014). Factors affecting treatment outcomes in drug-resistant tuberculosis cases in the Northern Cape, South Africa. Public Health Action.

[11] Kuchukhidze G, Kumar AMV, de Colombani P, Khogali M, Nanava U, Blumberg HM (2014). Risk factors associated with loss to follow-up among multidrug-resistant tuberculosis patients in Georgia. Public Health Action.

[12] Yunusbaeva M, Borodina L, Alekseev P, Davydov R, Yunusbaev U, Sharipov R (2019). Treatment efficacy of drug-resistant tuberculosis in Bashkortostan, Russia: a retrospective cohort study. Int J Infect Dis.

[13] O’Donnell MR, Zelnick J, Werner L, Master I, Loveday M, Horsburgh CR, et al. Extensively drug-resistant tuberculosis in women, KwaZulu-Natal, South Africa. Emerg Infect Dis J. 2011;17(10). https://wwwnc.cdc.gov/eid/article/17/10/11-0105_article. 10.3201/eid1710.110105 PMC3310667 22000378

[14] Sia D, Onadja Y, Hajizadeh M, Heymann SJ, Brewer TF, Nandi A (2016). What explains gender inequalities in HIV/AIDS prevalence in sub-Saharan Africa? Evidence from the demographic and health surveys. BMC Public Health.

[15] Hegdahl HK, Fylkesnes KM, Sandøy IF (2016). Sex differences in HIV prevalence persist over time: evidence from 18 countries in Sub-Saharan Africa. PLoS ONE.

[16] Pepper DJ, Schomaker M, Wilkinson RJ, de Azevedo V, Maartens G (2015). Independent predictors of tuberculosis mortality in a high HIV prevalence setting: a retrospective cohort study. AIDS Res Ther.

[17] Heunis JC, Kigozi NG, Chikobvu P, Botha S, van Rensburg HD (2017). Risk factors for mortality in TB patients: a 10-year electronic record review in a South African province. BMC Public Health.

[18] Okethwangu D, Birungi D, Biribawa C, Kwesiga B, Turyahabwe S, Ario AR (2019). Multidrug-resistant tuberculosis outbreak associated with poor treatment adherence and delayed treatment: Arua District, Uganda, 2013–2017. BMC Infect Dis.

[19] Baluku JB, Mugabe P, Mulwana R, Nassozi S, Katuramu R, Worodria W (2020). High prevalence of rifampicin resistance associated with rural residence and very low bacillary load among TB/HIV-coinfected patients at the national tuberculosis treatment center in Uganda. BioMed Res Int.

[20] Baluku JB, Nakazibwe B, Naloka J, Nabwana M, Mwanja S, Mulwana R (2021). Treatment outcomes of drug resistant tuberculosis patients with multiple poor prognostic indicators in Uganda: a countrywide 5-year retrospective study. J Clin Tuberc Mycobact Dis.

[21] Kasozi S, Kirirabwa NS, Kimuli D, Luwaga H, Kizito E, Turyahabwe S (2020). Addressing the drug-resistant tuberculosis challenge through implementing a mixed model of care in Uganda. PLoS ONE.

[22] Ministry of Health (2011). Uganda national guidelines for the programmatic management of drug resistant tuberculosis.

[23] Ministry of Health (2016). Uganda national guidelines for the programmatic management of drug resistant tuberculosis.

[24] Ministry of Health. Short treatment regimen (STR) for MDR-TB: Annex 9. In: Uganda national guidelines for the programmatic management of drug resistant tuberculosis. 2017.

[25] Baluku JB, Katuramu R, Naloka J, Kizito E, Nabwana M, Bongomin F (2021). Multidisciplinary management of difficult-to-treat drug resistant tuberculosis: a review of cases presented to the national consilium in Uganda. BMC Pulm Med.

[26] Ministry of Health (2016). Consolidated guidelines for prevention and treatment of HIV in Uganda.

[27] World Health Organization. Definitions and reporting framework for tuberculosis–2013 revision. World Health Organization; 2013. Report No.: 9241505346.

[28] Horton KC, MacPherson P, Houben RMGJ, White RG, Corbett EL (2016). Sex differences in tuberculosis burden and notifications in low- and middle-income countries: a systematic review and meta-analysis. PLoS Med.

[29] Wang M-G, Huang W-W, Wang Y, Zhang Y-X, Zhang M-M, Wu S-Q (2018). Association between tobacco smoking and drug-resistant tuberculosis. Infect Drug Resist.

[30] Zetola NM, Macesic N, Modongo C, Shin S, Ncube R, Collman RG (2014). Longer hospital stay is associated with higher rates of tuberculosis-related morbidity and mortality within 12 months after discharge in a referral hospital in Sub-Saharan Africa. BMC Infect Dis.

[31] Marçôa R, Ribeiro AI, Zão I, Duarte R (2018). Tuberculosis and gender—factors influencing the risk of tuberculosis among men and women by age group. Pulmonology.

[32] Mason PH, Snow K, Asugeni R, Massey PD, Viney K (2017). Tuberculosis and gender in the Asia-Pacific region. Aust N Z J Public Health.

[33] Ministry of Health. Uganda population-based HIV impact assessment (UPHIA) 2016–2017. Kampala: Ministry of Health; 2019. http://uac.go.ug/sites/default/files/UPHIA%20Final%20Report%20%5B2016%20-%202017%5D.pdf.

[34] Li W-B, Zhang Y-Q, Xing J, Ma Z-Y, Qu Y-H, Li X-X (2015). Factors associated with primary transmission of multidrug-resistant tuberculosis compared with healthy controls in Henan Province, China. Infect Dis Poverty.

[35] El Hamdouni M, Bourkadi JE, Benamor J, Hassar M, Cherrah Y, Ahid S (2019). Treatment outcomes of drug resistant tuberculosis patients in Morocco: multi-centric prospective study. BMC Infect Dis.

[36] Duraisamy K, Mrithyunjayan S, Ghosh S, Nair SA, Balakrishnan S, Subramoniapillai J (2014). Does alcohol consumption during multidrug-resistant tuberculosis treatment affect outcome?. Ann Am Thorac Soc.

[37] Pradipta IS, van’t Boveneind-Vrubleuskaya N, Akkerman OW, Alffenaar JC, Hak E (2019). Treatment outcomes of drug-resistant tuberculosis in the Netherlands, 2005–2015. Antimicrob Resist Infect Control.

[38] Atif M, Bashir A, Ahmad N, Fatima RK, Saba S, Scahill S (2017). Predictors of unsuccessful interim treatment outcomes of multidrug resistant tuberculosis patients. BMC Infect Dis.

[39] Keshavjee S, Gelmanova I, Shin S, Mishustin S, Andreev Y, Atwood S (2012). Hepatotoxicity during treatment for multidrug-resistant tuberculosis: occurrence, management and outcome. Int J Tuberc Lung Dis.

[40] Ali MH, Alrasheedy AA, Kibuule D, Godman B, Hassali MA, Ali HMH (2019). Assessment of multidrug-resistant tuberculosis (MDR-TB) treatment outcomes in Sudan; findings and implications. Expert Rev Anti Infect Ther.

[41] Kliiman K, Altraja A (2009). Predictors of poor treatment outcome in multi- and extensively drug-resistant pulmonary TB. Eur Respir J.

[42] Wen C-P, Chan T-C, Chan H-T, Tsai M-K, Cheng T-Y, Tsai S-P (2010). The reduction of tuberculosis risks by smoking cessation. BMC Infect Dis.

[43] Thomas B, Watson B, Senthil E, Deepalakshmi A, Balaji G, Chandra S (2017). Alcohol intervention strategy among tuberculosis patients: a pilot study from South India. Int J Tuberc Lung Dis.

[44] Kinai E, Hanabusa H (2005). Renal tubular toxicity associated with tenofovir assessed using urine-beta 2 microglobulin, percentage of tubular reabsorption of phosphate and alkaline phosphatase levels. AIDS Lond Engl.

[45] Perumal R, Abdelghani N, Naidu N, Yende-Zuma N, Dawood H, Naidoo K (2018). Risk of nephrotoxicity in patients with drug-resistant tuberculosis treated with kanamycin/capreomycin with or without concomitant use of tenofovir-containing antiretroviral therapy. J Acquir Immune Defic Syndr 1999.

[46] Giannini EG, Testa R, Savarino V (2005). Liver enzyme alteration: a guide for clinicians. CMAJ Can Med Assoc J.

[47] Metushi I, Uetrecht J, Phillips E (2016). Mechanism of isoniazid-induced hepatotoxicity: then and now. Br J Clin Pharmacol.

[48] Sharma SK (2010). Zidovudine-induced anaemia in HIV/AIDS. Indian J Med Res.

[49] Rushton DH, Dover R, Sainsbury AW, Norris MJ, Gilkes JJH, Ramsay ID (2001). Why should women have lower reference limits for haemoglobin and ferritin concentrations than men?. BMJ.

[50] Castelnuovo B, Mubiru F, Kalule I, Kiragga A (2019). Reasons for first line ART modification over the years during the ART scale up in Uganda. AIDS Res Ther.

[51] Azeez A, Ndege J, Mutambayi R (2018). Associated factors with unsuccessful tuberculosis treatment outcomes among tuberculosis/HIV coinfected patients with drug-resistant tuberculosis. Int J Mycobacteriol.

[52] Umanah TA, Ncayiyana JR, Nyasulu PS (2015). Predictors of cure among HIV co-infected multidrug-resistant TB patients at Sizwe Tropical Disease Hospital Johannesburg, South Africa. Trans R Soc Trop Med Hyg.

[53] Prezzemolo T, Guggino G, La Manna MP, Di Liberto D, Dieli F, Caccamo N (2014). Functional signatures of human CD4 and CD8 T cell responses to *Mycobacterium tuberculosis*. Front Immunol.

[54] Baluku J, Musaazi J, Mulwana R, Mugabo A, Bongomin F, Katagira W (2020). Prevalence and predictors of CD4+ T-lymphocytopenia among HIV-negative tuberculosis patients in Uganda. Res Rep Trop Med.

