# Correction: Gender differences among patients with drug resistant tuberculosis and HIV co-infection in Uganda: a countrywide retrospective cohort study

**DOI:** 10.1186/s12879-023-08014-4

**Published:** 2023-01-23

**Authors:** Joseph Baruch Baluku, David Mukasa, Felix Bongomin, Anna M. Stadelman, Edwin Nuwagira, Sabine Haller, Kauthrah Ntabadde, Stavia Turyahabwe

**Affiliations:** 1 Division of Pulmonology, Kiruddu National Referral Hospital, Kampala, Uganda; 2 Makerere University Lung Institute, PO Box 26343, Kampala, Uganda; 3 Complex Diseases and Genome Epidemiology, Graduate School of Public Health, Seoul National University, Seoul, South Korea; 4 Department of Medical Microbiology & Immunology, Faculty of Medicine, Gulu University, Gulu, Uganda; 5 Division of Epidemiology and Community Health, School of Public Health, University of Minnesota, Minneapolis, MN USA; 6 Infectious Diseases Unit, Department of Medicine, Mbarara University of Science and Technology, Mbarara, Uganda; 7 Department of Public and Global Health, Epidemiology, Biostatistics, & Prevention Institute, University of Zurich, Zurich, Switzerland; 8 MRC/UVRI & LSHTM Uganda Research Unit, Entebbe, Uganda; 9 National Tuberculosis and Leprosy Control Program, Ministry of Health, Kampala, Uganda

**Correction** **:** **BMC Infect Dis (2021) 21:1093** 10.1186/s12879-021-06801-5

Following publication of the original article [1], the authors identified an error in the author name of Anna M. Stadelman. Additionally, the ORCID number was previously unavailable.

The incorrect author name is: Anna Stadelmann

The correct author name is: Anna M. Stadelman

ORCID: 0000-0002-1457-0931

The author group and ORCID has been updated above and the original article [1] has been corrected.
